# Banana stem and leaf biochar as an effective adsorbent for cadmium and lead in aqueous solution

**DOI:** 10.1038/s41598-022-05652-7

**Published:** 2022-01-28

**Authors:** Xiyang Liu, Gaoxiang Li, Chengyu Chen, Xiaorui Zhang, Kuan Zhou, Xinxian Long

**Affiliations:** Guangdong Provincial Key Laboratory of Agricultural and Rural Pollution Abatement and Environmental Safety, College of Natural Resources and Environment, South China Agricultural University, 483 Wushan Road, Guangzhou, 510642 Guangdong China

**Keywords:** Environmental sciences, Materials science, Chemical physics

## Abstract

Lead (Pb) and cadmium (Cd) are toxic heavy metals commonly found in aqueous environments. Biochar as a green adsorbent generated from biomass feedstock may be used for effective removal of these heavy metals. This study investigated the adsorption kinetics and isotherms of Pb^2+^ and Cd^2+^ in aqueous solutions at different pH by biochar prepared from banana stem and leaf (BSL-BC) at 400 °C. Characterizations using scanning electron microscope, X-ray diffraction, and Fourier-transform infrared spectroscopy showed that the synthesized BSL-BC had rough surface, porous structure, and oxygen-containing functional groups. The adsorption of Pb^2+^ and Cd^2+^ onto BSL-BC reached equilibrium in 8 h and 200 min, respectively, with faster adsorption attained at higher pH and the optimum pH occurred at 5 (Pb^2+^) and 8 (Cd^2+^). All adsorption kinetic data followed the pseudo-second-order rate model. The adsorption isotherm data of Pb^2+^ and Cd^2+^ could be well-described by the Langmuir and Freundlich models, respectively, whereas neither the Temkin or Dubinin–Radushkevich models provided satisfactory fitting results. The maximum adsorption capacities for Pb^2+^ and Cd^2+^ were 302.20 and 32.03 mg/g, respectively. The calculated mechanism contributions showed that complexation with oxygen-containing functional groups, ion exchange, mineral precipitation, and Pb^2+^/Cd^2+^-π coordination accounted for 0.1%, 8.4%, 88.8%, and 2.6% to Pb^2+^ adsorption, and 0.4%, 6.3%, 83.0%, and 10.4% to Cd^2+^ adsorption, respectively. Therefore, mineral precipitation was likely the major mechanism responsible for adsorption of both Pb^2+^ and Cd^2+^ by BSL-BC. The results suggest that the synthesized BSL-BC has great potential for adsorption of Pb^2+^ and Cd^2+^ from aqueous solutions.

## Introduction

Heavy metals are widely present in aqueous environments due to water discharged from anthropogenic activities such as applications of fertilizer and pesticide, smelting and mining, and manufacture of electrical appliances, resulting in serious groundwater and surface water pollution^1^. Cadmium (Cd) and lead (Pb) are the most common and toxic heavy metals found in aqueous environments, posing acute and chronic effects on ecosystems and human health as they are persistent, migratable, bio-accumulative, and carcinogenic^2^. Therefore, it is important to treat water contaminated with heavy metals originated from domestic, agricultural, and industrial sources prior to its discharge into surface water or groundwater environments.

Heavy metals can be removed from wastewater and contaminated aqueous environments by various techniques, including reverse osmosis, ion-exchange, chemical precipitation, coagulation, electrochemical treatment, and physical adsorption^3–5^. Among them, adsorption is a promising approach for removing heavy metals from aqueous systems, with the benefits of cost-effectiveness, wide applicability, and ease of operation^6^. Various types of conventional (e.g., activated carbon, amorphous silica, clay minerals, diatomite, biochar, zeolites, and polymers) and novel nanosized (e.g., carbon nanotubes, graphene oxide, and reduced graphene oxide) adsorbents have been developed for metal treatment^4,7^. Compared with biochar, activated carbon and nanomaterials are relatively more expensive, while other conventional adsorbents such as natural zeolite/clay minerals generally have low adsorption efficiency; meanwhile, nanomaterials are difficult to be retrieved after adsorption of heavy metals^4^. Development of green adsorbents such as biochar from waste recycling that possess local availability, low cost, and high adsorption efficiency would be an environmental-friendly approach for heavy metal remediation.

Recent studies have highlighted biochar as an effective and green absorbent for removing heavy metals from water, due to its unique physicochemical properties including porous structure with abundant functional groups (e.g., carboxyl, carbonyl, and phenolic groups), large specific surface area (SSA), and readily available feedstock resources as raw material for adsorbent synthesis^8–10^. Biochar can be generated from incomplete combustion of organic matter under oxygen-limited conditions and at relatively low temperatures (i.e., 350–700 °C). Theoretically, biomass feedstock can include any organic waste materials, such as industrial wastes and by-products, forest and crop residues, algae, domestic solid wastes, sewage sludge, and manures^11^. Among different types of feedstocks, the derivation of biochar from crop residues (e.g., rice, corn, wheat straw, and woodchips) is gaining increasing attention as an approach for recycling agricultural wastes and achieving sustainable development^12^.

Banana is widely available and ranks the fourth most grown food crop worldwide after rice, wheat, and corn^13^. China is one of the countries with the longest history of banana cultivation. In 2017, the global banana production reached a record of 114 million tons, among which China accounted for ~ 10% (~ 11 million tons per year) and ranked the second largest banana producer^13^. However, banana fruit only weights ~ 12% of the whole plant, generating huge amounts (~ 220 t/hectare) of waste residues (i.e., stems, leaves, and rachis) during production^14^. Ortiz-Ulloa et al.^15^ reported that the average ratio of waste residue (i.e., above-ground biomass) to product (i.e., fruit) was 3.79, and that the biomass of stems and leaves contributed 78% and 17% to the above ground biomass, respectively. After harvesting banana fruits, the stems and leaves are usually abandoned in the field, taking months for natural degradation^16^, as the banana stem is primarily lignocellulosic in nature and mainly composed of 35–40% cellulose, 25–35% hemicellulose, and 8–13% lignin^17^. As banana stem and leaf (BSL) contain high lignin and low cellulose contents, they could be favorably considered as a raw material for producing biochar, which should ideally have a high production yield, large SSA, porous structure, and high fixed carbon content^18,19^. Such application can recycle the banana waste residues effectively for preparing adsorbents for treatment of heavy metals in contaminated water.

Therefore, this study prepared biochar from BSL (BSL-BC) via oxygen-limited pyrolysis under optimized conditions at 400 °C, and investigated the adsorption of Cd^2+^ and Pb^2+^ by the synthesized adsorbent in aqueous solutions. The objectives of this study were to: (1) characterize the physicochemical properties of BSL-BC and BSL; (2) examine and compare the adsorption rates and capacities of Cd^2+^ and Pb^2+^ onto BSL-BC in adsorption kinetic and isotherm experiments; (3) elucidate the adsorption mechanisms and quantify their relative contributions; and (4) compare the metal adsorption capacities of BSL-BC with other plant-based biochar with previous studies. The results would promote agricultural waste recycling and novel adsorbent development, as well as provide useful insights on adsorption mechanisms of heavy metals onto biochar.

## Materials and methods

### Preparation of adsorbent

The banana (*Musa acuminata*) stem and leaf sample was obtained from Dongguan city in Guangdong province of China (23° 3 N, 113° 5 E) with the consent of the crop owner, and was identified by Dr. Ping Li (South China Agricultural University) as banana (*Musa acuminata*). After washing thrice with double-distilled water, BSL was chopped to about 2-cm in length and dried at 80 °C for 24 h, and then milled to pass through a 0.154-mm sieve. Since the recovery rate of biochar after pyrolysis was ~ 50%, 50 g powdered BSL biomass was packed into a 304 stainless steel vessel, filled with nitrogen gas, and tightly capped, yielding ~ 25 g biochar in one pyrolysis cycle. The pyrolysis process of BSL for producing BSL-BC was conducted in a muffle furnace under the optimum pyrolysis condition of heating BSL at a rate of 10 °C/min to 400 °C, which was maintained for 3 h. The optimum pyrolysis conditions on temperature, residence time, and heating rate were determined from the experiments as described in S1 of the Supplementary Material, taking into considerations of both energy consumption and adsorption performance.

### Characterization of BSL and BSL-BC before adsorption

The chemical contents of BSL and BSL-BC were thoroughly characterized. The contents of total C, H, N, and P were measured with an elemental analyzer (Thermo Scientific FLASH 2000). The ash content was determined by the difference between the mass of 1 g BSL-BC heated at 750 °C for 6 h and the mass of the remaining material^20^. The total oxygen content (%) was determined by subtracting 100% by the contents (%) of ash, C, H, and N. The pH and electrical conductivity (EC) of BSL-BC were measured with a pH meter (Mettler Toledo 320-S) and a conductivity meter (DDB303A), respectively, by mixing BSL-BC with double-distilled water in a ratio 1:20 (w:v)^20^. The zeta potential of BSL-BC as a function of solution pH in double-distilled water was determined with a Zetasizer Nano ZS90 (Malvern, UK).

To determine the total contents of Pb and Cd on BSL-BC before adsorption, 0.5 g BSL-BC was digested with solution containing 7 mL HNO_3_, 3 mL HCL, and 3 mL HF. The mixture was sequentially heated in a microwave digester (ETHOS UP, Milestones Helping Chemists, Italy) at 130 °C for 5 min, 170 °C for 5 min, and 190 °C for 35 min. The Pb and Cd contents in the digested liquid were measured with a flame atomic absorption spectrophotometer (AAS, Z-2300, Hitachi, Japan).

The morphology and size of BSL and BSL-BC were characterized by scanning electron microscopy (SEM, Zeiss Sigma 300), with their element analysis conducted on the SEM equipped with energy dispersive X-ray spectrometry (SEM–EDS, Bruker Electric Cooling X-ray Spectrometer XFlash6). The SSA, total pore volume, and pore size distribution of BSL-BC were assessed by Brunauer–Emmett–Teller (BET) analysis using a NOVA 1200 surface area pore analyzer (Mike ASAP2020).

Fourier-transform infrared spectroscopy (FTIR) analysis was performed to determine the major organic functional groups on the surface of BSL-BC. The FTIR spectra between 400 and 4000 cm^−1^ for BSL-BC prepared in pellets of fused KBr were measured with Bruker Vector 22 spectrometer (PE FT-IR Frontier). The valance of specific elements was analyzed by energy dispersive X-ray spectroscopy (XPS, Thermo Fisher Scientific K-Alpha), and all binding energies were calibrated using C 1 s peak (284.8 eV). The crystallite phase composition was analyzed with powder X-ray diffraction (XRD), which was performed by an X’Pert PRO diffractor (BRUKER D8 Advance) with the tube parameters set at 40 kV of voltage and 40 mA of current.

### Adsorption experiments

The Cd^2+^ and Pb^2+^ adsorption experiments were performed using a batch equilibration technique for triplicate samples at room temperature. Stock solutions of Cd^2+^ and Pb^2+^ at 1000 mg/L were prepared with CdCl_2_·2.5H_2_O and Pb(NO_3_)_2_ in double-distilled water, respectively. The equilibrium adsorption amounts (*Q*_e_, mg/g) of Cd^2+^ or Pb^2+^ onto BSL-BC and the adsorption efficiencies (%) were calculated using Eqs. (1) and (2), respectively:

1 $$Q_{e} = \frac{{\left( {C_{0} - C_{e} } \right)V}}{M}$$

2 $$Adsorption\; efficiency\% = \frac{{C_{0} - C_{e} }}{{C_{0} }} \times 100\%$$

where *C*_0_ and *C*_e_ are the initial and equilibrium aqueous concentrations of Cd^2+^ or Pb^2+^ (mg/L), respectively, *V* is the solution volume (mL), and *M* is the mass of adsorbent (g).

#### Adsorption kinetics

To investigate the adsorption rate, 40 mg BSL-BC was mixed with 50 mL solution containing 200 mg/L Pb^2+^ at an initial pH of 5.0 ± 0.1 (unadjusted) or 50 mg/L Cd^2+^ at an initial pH of 5.5 ± 0.1 (unadjusted) in 150 mL conical flasks. The mixture was agitated at 180 rpm on a reciprocating shaker at 25 °C. Replicate flasks containing Pb^2+^ or Cd^2+^ were sampled at regular time intervals (5–1440 min), and filtered with 0.30–0.50 μm Double Ring quantitative filter paper. The filtrate was acidified with 1% (v/v) HNO_3_ (Guaranteed reagent, GR) and the concentration of Pb^2+^ or Cd^2+^ was determined by AAS (Z-2300, Hitachi, Japan).

The pseudo-first-order (PFO) (Eq. 3) and pseudo-second-order (PSO) (Eq. 4) kinetic models are two most frequently used models for fitting the adsorption rate data of metal ions^21^. We also investigated the rate-limiting step of adsorption by fitting the adsorption rate data with an intra-particle diffusion model (Eq. 5)^22^.

3 $$\ln Q_{e} - Q_{t} = lnQ_{e} - K_{1} t$$

4 $$\frac{t}{{Q_{t} }} = \frac{t}{{Q_{e} }} + \frac{1}{{K_{2} Q_{e}^{2} }}$$

5 $$Q_{t} = K_{d} t^{1/2} + I$$

where *Q*_t_ (mg g^−1^) is the amount adsorbed at time *t* (min), *K*_1_ (min^−1^) and *K*_2_ (g mg^−1^ min^−1^) are the PFO and PSO rate constants, respectively, *K*_d_ (mg g^−1^ h^−1/2^) is the rate constant of the intra-particle diffusion model, and *I* (mg g^−1^) is a constant corresponded to the boundary layer thickness. The values of *K*_d_ and *I* were obtained from the slope and intercept of the second linear regime of the intra-particle diffusion model, respectively.

#### Adsorption isotherms

To initiate the adsorption isotherm experiments, 50 mL solution with different initial concentrations of Pb^2+^ (10, 50, 100, 200, 300, 400, 500, 600, and 700 mg/L) or Cd^2+^ (10, 25, 50, 75, 100, 125, 150, and 200 mg/L) was added into a series of conical flasks, followed by addition of 40 mg BSL-BC. Adsorption experiments were conducted at initial pH values of 5.0 for Pb^2+^ and 5.5 for Cd^2+^. The mixture was shaken at 180 rpm and 25 °C for 8 h, after which it was filtered with 0.30–0.50 μm Double Ring quantitative filter paper, and the concentrations of Pb^2+^ and Cd^2+^ in the filtrate were analyzed by AAS (Z-2300, Hitachi, Japan).

The adsorption isotherm data were fitted with the Langmuir (Eq. 6), Freundlich (Eq. 7), Temkin (Eq. 8), and Dubinin–Radushkev (D–R) (Eq. 9) isotherm models:

6 $$\frac{1}{{Q_{e} }} = \frac{1}{{K_{l} C_{e} Q_{m} }} + \frac{1}{{Q_{m} }}$$

7 $$Q_{e} = K_{f} C_{e}^{1/n}$$

8 $$Q_{e} = BlogA + BlogC_{e}$$

9 $$lnQ_{e} = lnQ_{m} - K\varepsilon^{2}$$

10 $$B = \frac{RT}{b}$$

11 $$\varepsilon = RT\cdot{\text{ln}}\left( {1 + 1/C_{e} } \right)$$

where *Q*_m_ (mg g^−1^) is the maximum adsorption amount, *K*_l_ (L mg^−1^) is the Langmuir model constant, *K*_f_ (L g^−1^) is the Freundlich model constant, *n* is a constant related to the adsorption strength, *R* is the gas constant (8.314 (J mol^−1^ K^−1^), *T* is absolute temperature (K), *b* is the Temkin constant related to the adsorption heat (J mol^−1^), *A* is the Temkin isotherm constant (L g^−1^), *K* is the D–R isotherm parameter used for estimating the mean free energy (*E* = $1/\sqrt {2K}$) to distinguish the type of adsorption process^23^, and *ε* is the D–R isotherm parameter.

#### Effects of pH

The pH effect on Pb^2+^ adsorption was studied by mixing 50 mg BSL-BC and 50 mL solutions containing 200 mg/L Pb^2+^ at different initial pH (2.0–6.0) in 100 mL centrifuge tubes, as Pb^2+^ may precipitate as hydroxides at pH ≥ 7^24^. To investigate the pH effect on Cd^2+^ adsorption, 50 mg BSL-BC was mixed with 50 mL solutions containing 50 mg/L Cd^2+^ at different initial pH (2.0–8.0). The solution pH was adjusted with 0.1 M HCl or NaOH before adding BSL-BC. After shaken at 180 rpm and 25 °C for 8 h, the mixture was filtered with 0.30–0.50 μm Double Ring quantitative filter paper, and the concentrations of Pb^2+^ and Cd^2+^ in the filtrate were analyzed by AAS (Z-2300, Hitachi, Japan).

### Characterization of BSL-BC after adsorption

To investigate the adsorption mechanisms of Pb^2+^or Cd^2+^ onto BSL-BC, 50 mg BSL-BC was added into 50 mL solutions containing 200 mg/L Pb^2+^ (pH 5.0) or 50 mg/L Cd^2+^ (pH 5.6) in 150 mL conical flasks. The mixture was shaken at 180 rpm and 25 °C for 8 h. After filtration, the BSL-BC samples loaded with Pb^2+^ or Cd^2+^ were recovered and dried at 40 °C. The changes in morphology and functional groups of BSL-BC after adsorption were characterized by SEM–EDS, FTIR, and XRD.

### Determination of mechanism contributions to adsorption

The contribution of different mechanisms to Pb^2+^/Cd^2+^ adsorption onto BSL-BC was calculated based on the modified method proposed by Wang et al.^20^ and Cui et al.^25^. Firstly, BSL-BC was demineralized by soaking for 30 min in 1 M HCl, rinsed with double-distilled water until stable solution pH, air-dried, and weighed. The demineralization rate, *Y* (%), was calculated according to the mass before and after demineralization. Fifty milligrams of the original BSL-BC or the demineralized BSL-BC were weighted into a 150 mL triangular flask containing 50 mL of 50 mg/L Cd^2+^ or 200 mg/L Pb^2+^. The mixture was shaken at 180 rpm and 25 °C for 8 h, and then filtered through Double Ring quantitative filter paper. The filtrate was collected for analysis of concentrations for Pb^2+^, Cd^2+^, K^+^, Na^+^, Ca^2+^, and Mg^2+^ by AAS (Z-2300, Hitachi, Japan). Double-distilled water was used as a control group.

The adsorption capacities attributed to complexation with oxygen-containing functional group (*Q*_co_), metal ion exchange (*Q*_cme_), mineral precipitation (*Q*_cmp_), and Pb^2+^/Cd^2+^-π coordination (*Q*_cπ_) were determined as follows:

12 $$Q_{cm} = Q_{t1} - Q_{a} *Y$$

13 $$Q_{cme} = \frac{1}{2}Q_{K} + Q_{Ca} + \frac{1}{2}Q_{Na} + Q_{Mg}$$

14 $$Q_{cmp} = Q_{cm} - Q_{cme}$$

15 $$Q_{co} = Q_{co1} *Y$$

16 $$Q_{c\pi } = Q_{a} *Y - Q_{co}$$

where *Q*_cm_ is the amount of Pb^2+^/Cd^2+^ adsorption attributed to interaction with minerals (mg/g), *Q*_t1_ and *Q*_a_ are the total adsorption capacities before and after demineralization (mg/g), respectively, and *Q*_K_, *Q*_Na_, *Q*_Ca_, and *Q*_Mg_ are the amounts of cations (K^+^, Na^+^, Ca^2+^, and Mg^2+^, respectively) released from biochar (mg/g) into Pb^2+^/Cd^2+^ solution after subtracting those leached into double-distilled water. Since the amount of H^+^ released could be determined by the decrease of pH, the unadjusted adsorbed amount of Pb^2+^ or Cd^2+^ via complexation with oxygen-containing functional group (*Q*_co1_, mg/g) was calculated accordingly, which was multiplied by *Y* to offset the concentration effect.

## Results and discussion

### Characteristics of BSL and BSL-BC

Both BSL and BSL-BC were characterized to examine the basic properties of the raw material and to reveal the change in properties after preparation into biochar. The major characteristics of the raw material (BSL) and synthesized adsorbent (BSL-BC) are presented in Table 1, with their photos presented in Fig. S1a,b, respectively. Compared with BSL, BSL-BC had higher contents of C, O, and N but lower H content. The molar ratios of H/C and O/C on BSL-BC were about 0.058 and 0.34, respectively, indicating that BSL-BC had high aromaticity and hydrophobicity^26^. The cation contents for K^+^, Na^+^, Ca^2+^, and Mg^2+^ on BSL-BC were 5.91, 1.26, 0.37, and 5.71 mg/g, respectively. The digestion analysis shows that before adsorption, BSL-BC had trace amounts of Cd (0.0054 mg/g) and Pb (0.10 mg/g).

**Table 1:** Physicochemical properties of BSL and BSL-BC before adsorption.

Sample	pH	EC ^a^	SSA ^b^	Pore volume	Pore diameter	Ash content	Elements (%)					Heavy metals (mg/kg)		Other cations (mg/g)			
		(μS/cm)	(m^2^/g)	(cm^3^/g)	(nm)	(%)	C	H	N	O	P	Cd	Pb	K	Ca	Na	Mg
BSL	–	–	0.7860	0.002000	9.309	–	42.34	5.520	1.080	–	0.0004200	–	–	–	–	–	–
BSL-BC	9.980	5.520	15.73	0.06800	17.04	17.27	58.19	3.380	1.380	19.78	0.001000	0.005400	0.1000	5.910	1.260	0.3700	5.710

^a^*EC* Electrical conductivity.

^b^*SSA* Specific surface area.

The double-distilled water containing only BSL-BC had solution pH of 10.2. Fig. S2 shows that BSL-BC remained negatively charged under most pH conditions in double-distilled water, yielding a point of zero charge (PZC) at pH 1.2. As shown in the FTIR spectrum (Fig. S3), the surface of BSL-BC mainly contained C=C (1320 cm^−1^) and –CH (780 cm^−1^) as well as oxygen-containing functional groups including –OH (3430 cm^−1^) and C=O (1615 cm^−1^), which would contribute to the adsorption of positively charged heavy metals such as Pb^2+^ and Cd^2+^. The hydroxyl and carboxylic groups should be responsible for the deprotonation of BSL-BC in water that resulted in its negatively charged surface.

The SEM image (Fig. S4a) shows that the surface of BSL was covered with cracks and it had irregular lamellar structures stacked in layers. After pyrolysis, BSL-BC displayed many wrinkles and irregular pore-like structures uniformly distributed on the surface (Fig. S4b). The inner walls of these pores in BSL-BC were relatively smooth, which may provide surface area for adsorption. Table 1 shows that BSL-BC had larger SSA, greater total pore volume, and smaller average pore diameter (15.73 m^2^/g, 0.06800 cm^3^/g, and 17.04 nm, respectively) than BSL (0.7860 m^2^/g, 0.002000 cm^3^/g, and 9.309 nm, respectively), indicating that BSL-BC could provide more available sites for adsorption and storage of metal ions^1^. The SSA of BSL-BC (15.73 m^2^/g) was similar to the SSA values (1–50 m^2^/g) reported in the literature for biochar made from eucalyptus sawdust^27^, activated sludge, cow biosolids^28^, and banana peel^29^; however, it was significantly smaller than the SSA values (50–500 m^2^/g) for biochar made from other plant residues such as charcoal, sugarcane bagasse, rape straw, wheat straw, Miscanthus straw, and soft wood^26,30^.

### Adsorption kinetics

**Figure 1:**
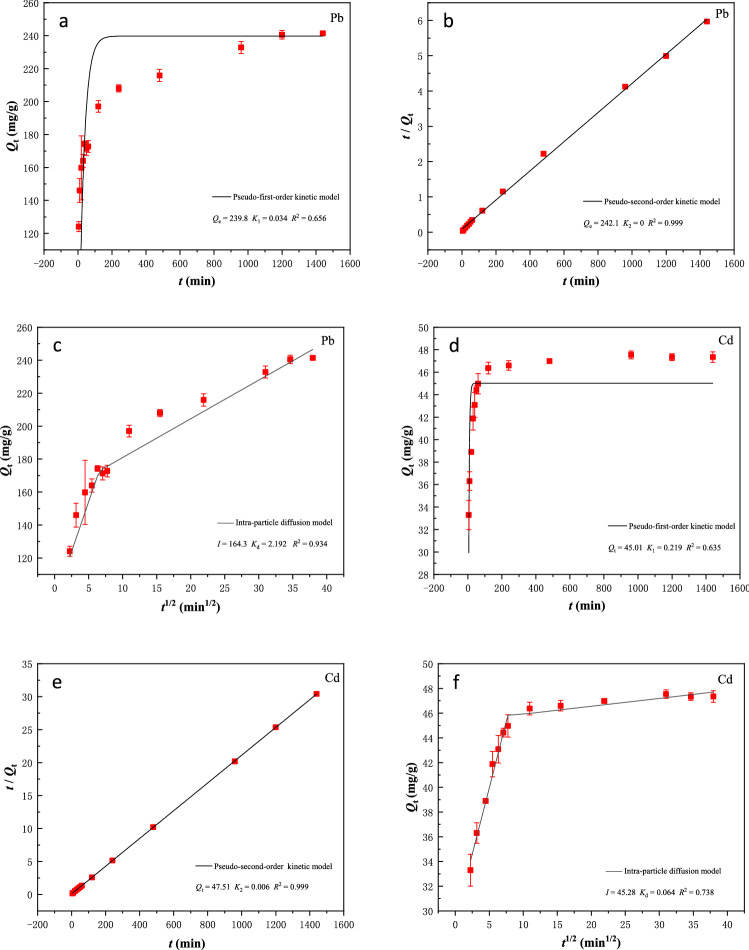
Adsorption kinetic data of Pb^2+^ onto BSL-BC fitted by the (**a**) PFO and (**b**) PSO kinetic models and (**c**) intra-particle diffusion model. Adsorption kinetic data of Cd^2+^ onto BSL-BC fitted by the (**d**) PFO and (**e**) PSO kinetic models and (**f**) intra-particle diffusion model. The solutions containing 0.8 g/L BSL-BC and initial concentrations of 200 mg/L Pb^2+^ (pH 5.0) or 50 mg/L Cd^2+^ (pH 5.5) were mixed at 180 rpm and 25 °C.

The adsorption kinetics of 200 mg/L Pb^2+^ at pH 5.0 and 50 mg/L Cd^2+^ at pH 5.5 by 0.8 g/L BSL-BC within the first 24 h are presented in Fig. 1. As shown in Fig. 1a, high adsorption rate of Pb^2+^ onto BSL-BC was observed in the first 5 min and then gradually decreased after 200 min of contact. The adsorption amounts of Pb^2+^ still slowly increased until ~ 960 min, when adsorption equilibrium was attained. However, BSL-BC adsorbed Cd^2+^ rapidly in 200 min and approached equilibrium (Fig. 1d). The adsorption of Pb^2+^ appeared to be faster than Cd^2+^ by BSL-BC, as 63.4% of 200 mg/L Pb^2+^ was rapidly removed within the first 5 min, during which only 6.3% of 50 mg/L Cd^2+^ was removed from solution. At this stage, the adsorbed amounts of Pb^2+^ and Cd^2+^ onto BSL-BC were 152.8 and 3.140 mg/g, respectively, indicating that the adsorption capacity of BSL-BC for Pb^2+^ was much stronger than that for Cd^2+^.

The adsorption kinetic data of both Pb^2+^ (Fig. 1b) and Cd^2+^ (Fig. 1e) onto BSL-BC could be well-described by the PSO model, showing high regression coefficients of *R*^2^ = 0.999 for both Pb^2+^ and Cd^2+^ (Table 2). Meanwhile, the theoretical *Q*_e_ values fitted by PSO model were 239.8 and 45.01 mg/g for Pb^2+^ and Cd^2+^, respectively, which were consistent with their experimental results of 242.1 and 47.51 mg/g (Table 2). In comparison, the PFO model provided less ideal fitting for both metal ions (Fig. 1a–d), with *R*^2^ of 0.656 and 0.635 for Pb^2+^ and Cd^2+^, respectively. The better fitting results of PSO model than the PFO model indicated that chemical adsorption was mainly responsible for the removal of Pb^2+^ and Cd^2+^ by BSL-BC^31^.

**Table 2:** Fitting parameters of adsorption kinetic models for the adsorption of Pb^2+^ and Cd^2+^ onto BSL-BC.

Kinetic model	Fitting parameter	Pb^2+^	Cd^2+^
Pseudo-first-order	*Q*_e_ (mg g^−1^)	239.8	45.01
	*K*_1_	0.034	0.219
	*R*^2^	0.656	0.635
Pseudo-second-order	*Q*_e_ (mg g^−1^)	242.1	47.51
	*K*_2_	0	0.006
Intra-particle diffusion	*R*^2^	0.999	0.999
	*K*_d_ (mg g^−1^ h^−1/2^)	2.192	0.064
	*I* (mg g^−1^)	164.3	45.28
	*R*^2^	0.934	0.738

The values of *K*_d_ and *I* were obtained from the slope and intercept of the second linear regime of the intra-particle diffusion model, respectively. The solutions containing 0.8 g/L BSL-BC and initial concentrations of 200 mg/L Pb^2+^ (pH 5.0) or 50 mg/L Cd^2+^ (pH 5.5) were mixed at 180 rpm and 25 °C.

The intra-particle diffusion model was further fitted to the kinetic data for investigating the rate-limiting step of adsorption. The results show that the adsorption of both Pb^2+^ and Cd^2+^ on BSL-BC could be divided into two linear regimes, with the first regime showing a steeper slope than the second one. This suggests that the adsorption proceeded through two steps: the first linear regime describes a fast bulk diffusion step due to boundary effects, whereas the second regime describes a slow equilibrium attainment due to intra-particle diffusion processes^2^. The fitted correlation coefficients of *R*^2^ for adsorption of Pb^2+^ (0.934) was larger than Cd^2+^ (0.738), indicating that the intra-particle diffusion model was more suitable for describing the adsorption process of Pb^2+^ onto BSL-BC than Cd^2+^ (Table 2). The larger *K*_d_ value of Pb^2+^ (2.192 mg g^−1^ h^−1/2^) than that Cd^2+^ (0.064 mg g^−1^ h^−1/2^) suggested a faster diffusion of Pb^2+^ into the porous structure of BSL-BC. Meanwhile, the greater *I* value of Pb^2+^ (164.3 mg/g) than Cd^2+^ (45.28 mg/g) indicated that the adsorption of Pb^2+^ by BSL-BC experienced a stronger boundary layer effect (i.e., molecular diffusion in solution) than Cd^2+^ ^32^.

### Adsorption isotherms

**Figure 2:**
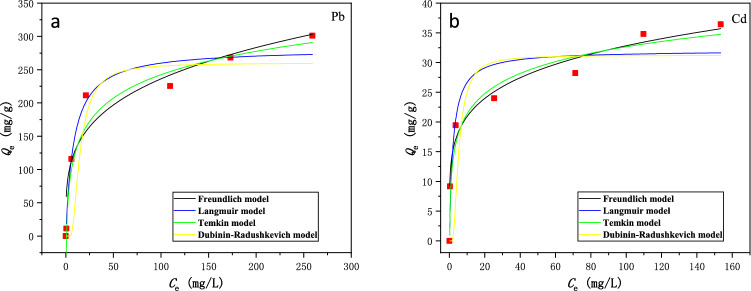
Adsorption isotherms of (**a**) Pb^2+^ and (**b**) Cd^2+^ on BSL-BC fitted with 4 isotherm models. The solutions containing 1 g/L BSL-BC and initial concentrations of 10–700 mg/L Pb^2+^ (pH 5.0) or 10–200 mg/L Cd^2+^ (pH 5.5) were mixed for 8 h at 180 rpm and 25 °C.

The adsorption isotherm experiments were conducted to investigate the equilibrium adsorption behaviors of Pb^2+^ and Cd^2+^ onto BSL-BC. Figure 2 shows that upon adsorption equilibrium, the solid phase adsorbed amount (*Q*_e_) of both metals increased drastically with their aqueous concentration (*C*_e_) at low *C*_e_ range. Such an increase became less significant at higher *C*_e_ likely due to adsorbent saturation. As the initial Pb^2+^ aqueous concentration (*C*_0_) increased from 10 to 500 mg/L, *Q*_e_ increased from 11.30 to 301.02 mg/g, with removal efficiencies decreasing from 94.1 to 46.4% (Fig. 2a). For Cd^2+^, *Q*_e_ increased from 9.17 to 28.23 mg/g and the removal efficiencies decreased from 97.5 to 28.4% as *C*_0_ increased from 10 to 200 mg/L (Fig. 2b). The results showed that the *Q*_e_ values for Pb^2+^ were an order of magnitude higher than those for Cd^2+^ under the tested conditions, indicating the stronger adsorption of BSL-BC for Pb^2+^ than Cd^2+^. Since Pb could be classified as a hard Lewis acid compared with Cd, the hydroxyl and carboxyl groups (hard Lewis bases) on the adsorbent likely had higher affinity for Pb; in addition, the smaller hydration radius and lower p*K*_H_ (negative logarithm of the hydrolysis constant) of Pb compared with Cd may also contribute to the stronger adsorption of Pb, as previously shown for another adsorbent (MgBC400)^33^.

The Langmuir, Freundlich, Temkin, and D–R isotherm models widely adopted for evaluation of adsorption behaviors^21,34^ were used to fit the isotherm data (Fig. 2). The fitting parameters for each isotherm model are compiled in Table 3. All 4 isotherm models could well-describe the adsorption of Pb^2+^ by BSL-BC, with the Langmuir model showing the best fit (*R*^2^ = 0.961) followed by Temkin (*R*^2^ = 0.957), D–R (*R*^2^ = 0.713), and Freundlich (*R*^2^ = 0.900) models (Fig. 2a and Table 2). However, the Freundlich (*R*^2^ = 0.968) and Temkin (*R*^2^ = 0.936) models provided better fitting results to the adsorption of Cd^2+^ onto BSL-BC as compared with the Langmuir (*R*^2^ = 0.791) and D–R (*R*^2^ = 0.437) models (Fig. 2b and Table 3). As shown in Fig. 2, the adsorption isotherms of both Pb^2+^ and Cd^2+^ onto BSL-BC displayed similar “L” type shapes.

**Table 3:** Fitting parameters of adsorption isotherm models for the adsorption of Pb^2+^ and Cd^2+^ onto BSL-BC.

Isotherm model	Fitting parameter	Pb^2+^	Cd^2+^
Langmuir	*Q*_m_ (mg g^−1^)	302.2	32.03
	*K*_1_ (L mg^−1^)	0.062	0.667
	*R*^2^	0.961	0.791
Freundlich	*K*_f_ (L g^−1^)	62.35	12.96
	1/*n*	0.272	0.192
	*R*^2^	0.900	0.968
Temkin	*A* (L g^−1^)	1.260	15.91
	*b* (J mol^−1^)	21.52	247.6
	*R*^2^	0.747	0.673
Dubinin–Radushkevich	*Q*_m_ (mg g^−1^)	217.9	28.04
	*K*^2^ (mol^2^ J^−2^)	5.040 × 10^–7^	6.888 × 10^–8^
	*E* (kJ mol^−1^)	1.000	2.694
	*R*^2^	0.713	0.437

The solutions containing 1 g/L BSL-BC and initial concentrations of 10–700 mg/L Pb^2+^ (pH 5.0) or 10–200 mg/L Cd^2+^ (pH 5.5) were mixed for 8 h at 180 rpm and 25 °C.

Since the Langmuir model provided the best fit on Pb^2+^ adsorption by BSL-BC, the above result indicates that monolayer adsorption of Pb^2+^ occurred at homogeneous sites with equal energy on BSL-BC^35–37^. On the other hand, according to the assumptions of Freundlich model, multilayer adsorption of Cd^2+^ should have taken place on heterogeneous surface with different binding energies on BSL-BC^35,38–41^. The values of 1/*n* obtained from the Freundlich model were 0.272 and 0.192 for Pb^2+^ and Cd^2+^, respectively (Table 3). This parameter represents the relative distribution of energy sites and relates to the favorable level of the adsorption system. For instance, the adsorption is generally pseudo-irreversible when 1/*n* < 0.01, strongly favorable between 0.01 and 0.1, favorable between 0.1 and 0.5, pseudo-reversible between 0.5 and 1, and unfavorable when above 1^42^. Therefore, the results imply that both the adsorption of Pb^2+^ and Cd^2+^ by BSL-BC was favorable.

The Temkin model assumes a uniform distribution of binding energies at the adsorbent surface, and that the adsorption heat decreases linearly with *B* in Eq. (8) rather than logarithmically owing to sorbate-sorbent interactions^21^. The fitting results in Table 3 demonstrate that the Temkin model did not provide satisfactory fitting to the adsorption isotherm data. Low correlation coefficients (*R*^2^) were obtained for Pb^2+^ (0.747) and Cd^2+^ (0.673). Meanwhile, the Temkin isotherm constants (*b*) (Eq. 10) of 21.52 and 247.6 J/mol for Pb^2+^ and Cd^2+^, respectively, describing the adsorption heat were also quite low^43^.

The D–R isotherm model generally applies to heterogeneous adsorbent surfaces^44^, and can be used to estimate the free energy, apparent porosity, and biosorption characteristics^45^. The biosorption mean free energy (*E*) calculated from the D–R isotherm model provides insights on the biosorption mechanism. The biosorption is a chemical process via ion exchange if *E* is 8–16 kJ/mol, and is a physical process if *E* < 8 kJ/mol^46^. The fitted *E* values for Pb^2+^ and Cd^2+^ were 1.000 and 2.694 kJ/mol, respectively (Table 3), indicating that the adsorption a physical process. This contradicts with the earlier PSO adsorption kinetic model fitting results that the adsorption was a chemical process. Considering the low *R*^2^ of the D–R model fitting, the adsorption should be mainly a chemical process. The higher value of biosorption coefficient (*K*^2^) for Pb^2+^ (5.040 × 10^–7^ mol^2^ J^−2^) than Cd^2+^ (6.888 × 10^–8^ mol^2^ J^−2^) indicates that the free energy for adsorption of Pb^2+^ was larger than Cd^2+^ on BSL-BC.

The maximum monolayer adsorption capacities (*Q*_m_) obtained from the Langmuir model were 302.2 and 32.03 mg/g Cd^2+^ and Pb^2+^, respectively, which were slightly higher than those obtained by the D–R model (217.9 and 28.04 mg/g Cd^2+^ and Pb^2+^, respectively) (Table 3). The adsorption capacities of BSL-BC for Cd^2+^ and Pb^2+^ derived from the Langmuir model were compared with other biochar reported in the literature, with the experimental conditions given (Table S4). The results show that BSL-BC is an effective adsorbent for two heavy metals. Compared with other biochar, BSL-BC exhibited superior maximum adsorption capacity especially for Pb^2+^ (302.2 mg/g), which was higher than the camellia seed husk biochar (109.7 mg/g)^47^, peanut shell biochar (52.80 mg/g)^20^, and wheat straw biochar (100.00 mg/g)^36^. Meanwhile, the maximum adsorption capacity of Cd^2+^ by BSL-BC (32.03 mg/g) was also higher than the rice husk biochar (9.670 mg/g)^31^ and the wheat straw biochar (19.72 mg/g)^36^, yet lower than the dairy manure biochar (51.40 mg/g)^48^. Consistent with our above-mentioned results, other biochar materials in the literature also had stronger adsorption capacity for Pb^2+^ than Cd^2+^ (Table S4).

### Effects of solution pH on adsorption

**Figure 3:**
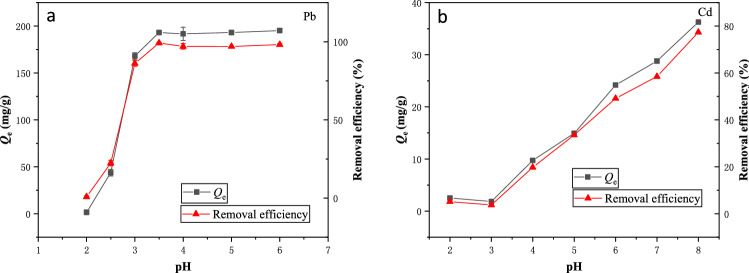
Effects of initial solution pH on the equilibrium adsorption amount (*Q*_e_) and removal efficiency of (**a**) Pb^2+^ and (**b**) Cd^2+^ by BSL-BC. The solutions containing 1 g/L BSL-BC and initial concentrations of 10–700 mg/L Pb^2+^ (pH 5.0) or 10–200 mg/L Cd^2+^ (pH 5.5) were mixed for 8 h at 180 rpm and 25 °C.

Figure 3 shows the variation of heavy metal adsorption onto BSL-BC with different initial solution pH. For both Pb^2+^ and Cd^2+^, their adsorption capacities and removal efficiencies by BSL-BC all greatly increased at higher pH. The most significant increase in Pb^2+^ adsorption onto BSL-BC occurred as pH increased from 2.0 to 3.5, after which the adsorption became steady with the maximum adsorption amount reaching 195.3 mg/g and removal efficiency approaching 100% at pH 6 (Fig. 3a). In comparison, low removal efficiency (< 5.2%) of Cd^2+^ by BSL-BC was observed at pH 2.0–3.0. Further increase in solution pH from 3.0 to 8.0 resulted in gradually enhancement of Cd^2+^ adsorption by BSL-BC, yielding the maximum adsorption amount and removal efficiency of 36.3 mg/g and 77.4% at pH 8.0, respectively (Fig. 3b).

The above results indicate that the initial solution pH strongly influenced both the adsorption of Pb^2+^ and Cd^2+^ by BSL-BC. The pH effects were mainly ascribed to their influence on the distribution of Pb^2+^ and Cd^2+^ in solution as well as the deprotonation state of surface functional groups on BSL-BC^49^. Pb^2+^ is the major species at solution pH < 7.5, with some fractions of lead present as Pb(OH)^+^ and PbHCO_3_^3+^ at pH > 5.0^47^. Similarly, Cd^2+^ also predominates in solution at pH 2.0–8.0 as Cd has low hydrolysis tendency at pH < 8.0^39^. Therefore, under acidic conditions (e.g., pH 2.0–3.0), abundant amounts of H^+^ in solution can effectively compete with Pb^2+^ and Cd^2+^ cations for active adsorption sites on the surface of BSL-BC^50^.

Meanwhile, Fig. S2 shows that with a PZC of 1.2, BSL-BC had low negative surface charge density under pH < 3.0. Its zeta potential changed from − 30 to − 40 mV as the solution pH increased from 3.0 to 4.0 and remained stable near − 40 mV at higher pH, which was due to deprotonation of functional groups such as –OH and –COOH as indicated by the FTIR spectra (Fig. S3). Consequently, in acidic solutions, weak electrostatic attraction existed between the adsorbent (BSL-BC) surface and the adsorbate (Pb^2+^ and Cd^2+^)^51^, with H^+^ also competing for adsorption sites, leading to the low removal efficiency. The results also indicate that electrostatic attraction and complexation with functional groups might be involved in the Pb^2+^ and Cd^2+^ adsorption process by BSL-BC.

### Adsorption mechanisms

The above adsorption kinetic and isotherm results (Figs. 1, 2, 3) have confirmed the effective adsorption of Pb^2+^ and Cd^2+^ onto BSL-BC. It is generally recognized that biochar adsorbs Pb^2+^ and Cd^2+^ via complexation with oxygen-containing functional groups (e.g., carboxyl and hydroxyl), cation exchange (e.g., with K^+^, Na^+^, Ca^2+^, and Mg^2+^), precipitation with minerals (e.g., CO_3_^2−^, PO_4_^3−^, and OH^−^), and coordination with π electrons^38,52–54^. The potential mechanisms responsible for the adsorption of Pb^2+^ and Cd^2+^ onto surface of BSL-BC were further explored at the microscale level based on FTIR, XPS, and XRD analyses, with the contribution of each mechanism quantified.

#### Functional group complexation

The FTIR spectra of BSL-BC before and after adsorption of Pb^2+^ or Cd^2+^ are presented in Fig. S3. The results show that the C–O absorption peak (1318 cm^−1^) was shifted to the right and the –OH peak (3430 cm^−1^) on BSL-BC was reduced significantly after contact with Pb^2+^. Similarly, these two peaks on Cd^2+^-loaded BSL-BC also obviously weakened. Since these oxygen-containing functional groups were consumed during the adsorption of Pb^2+^ and Cd^2+^, they likely participated in the complexation with the metal ions^31^.

**Figure 4:**
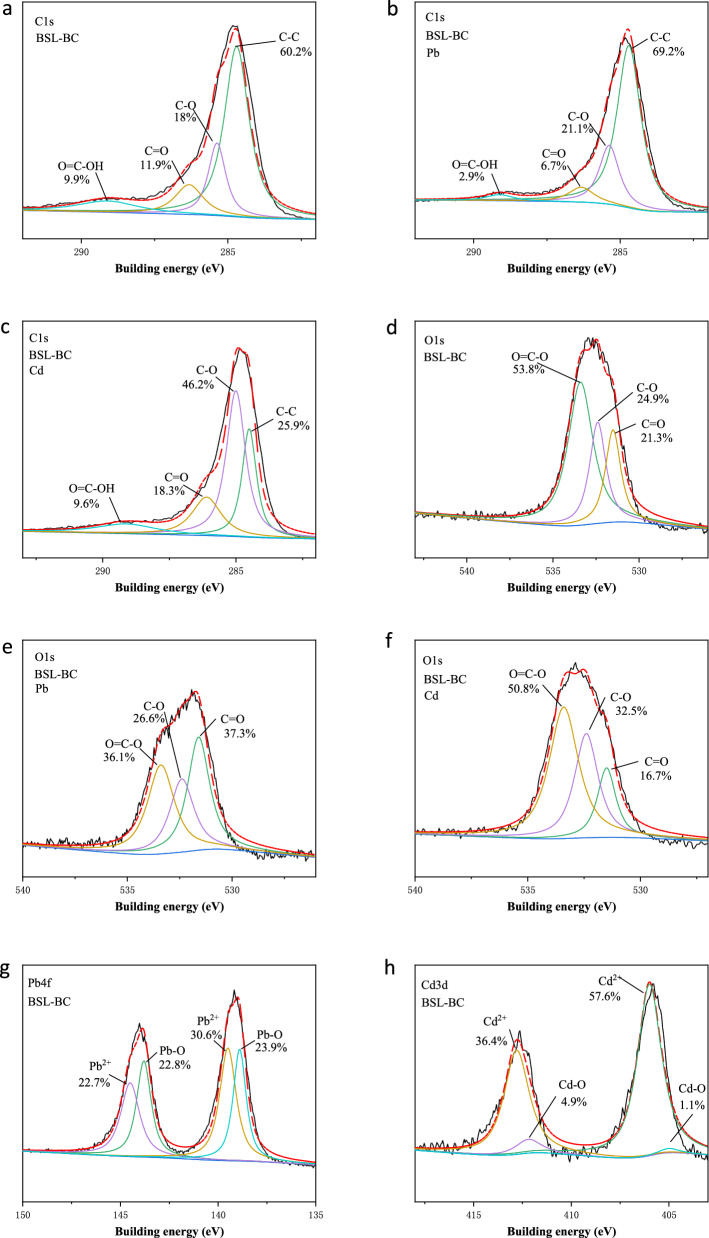
XPS spectra of elemental scan of C1s on BSL-BC (**a**) before and after adsorption of (**b**) Pb^2+^ or (**c**) Cd^2+^; XPS spectra of elemental scan of O1s on BSL-BC (**d**) before and after adsorption of (**e**) Pb^2+^ or (**f**) Cd^2+^; (**g**) XPS spectra of elemental scan of Pb4f. on BSL-BC after Pb^2+^ adsorption; (**h**) XPS spectra of elemental scan of Pb4f. on BSL-BC after Cd^2+^ adsorption.

The XPS results presented in Fig. 4 also confirmed with the FTIR results that complexation with oxygen-containing functional groups was responsible for the adsorption of both metals. For Pb^2+^, comparing the high-resolution C1s spectra of BSL-BC before (Fig. 4a) and after (Fig. 4b) adsorption shows that the peak area of –COOH decreased from 9.9 to 2.9% while the peak area of C–O increased slightly from 18 to 21.1%. This corresponds to the high-resolution O1s spectra before (Fig. 4d) and after adsorption of Pb^2+^ (Fig. 4e), which also suggest that the peak area of − COOH decreased from 53.8 to 36.1% while the peak area of C–O increased slightly from 24.9 to 26.6%. These results indicate that the adsorbed Pb^2+^ formed bidentate complexes (–O–Pb–O–) rather than monodentate complexes (–O–Pb–OH)^55^. Furthermore, the Pb4f. spectra after Pb^2+^ adsorption clearly identified the presence of Pb^2+^ (53.3%) and Pb–O (46.7%) on the surface of BSL-BC (Fig. 4g), since XPS analysis mainly probes the sample surface elements. The chemical formations may be due to the precipitation of Pb oxalate and Pb hydroxide during the adsorption of Pb^2+^.

For Cd^2+^, comparing the high-resolution C1s spectra of BSL-BC before (Fig. 4a) and after (Fig. 4c) adsorption shows that the peak area of C–O increased from 18.2 to 46.2%. Consistently, the C–O peak area in the O1s spectra also increased from 24.9 to 32.5% after Cd^2+^ adsorption (Fig. 4d,f). In addition, the Cd3d spectra after adsorption prove the presence of Cd^2+^ (94%) or Cd–O (6%) on the BSL-BC surface (Fig. 4h), which could be attributed to the precipitation of Cd carbonates (pebbles) and/or Cd hydroxides. The results indicate the involvement of C–O in the complexation process during the adsorption of Cd^2+^ and the formation of dentate complexes (–O–Cd–O–). Surface complexation of Pb^2+^ and Cd^2+^ with oxygen-containing functional groups (e.g., –OH and –COOH) has been suggested as a crucial mechanism for the adsorption of metal ions by biochar^38^. The above characterization results fully demonstrate that Cd^2+^ interacts with the oxygen-containing functional groups on the surface of BSL-BC during adsorption to form the CdCO_3_ complex.

#### Ion exchange

**Figure 5:**
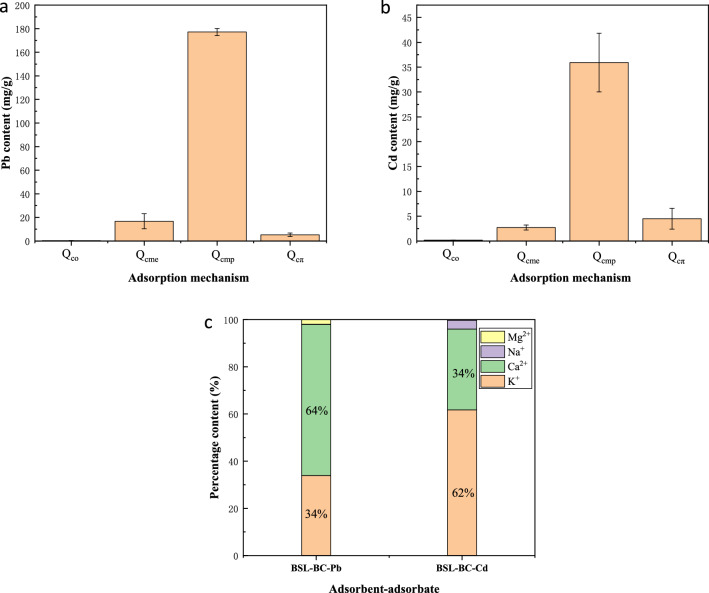
Mechanism contributions of complexation with oxygen-containing functional groups (*Q*_co_), metal ion exchange (*Q*_cme_), mineral precipitation (*Q*_cmp_), and Pb^2+^/Cd^2+^-π coordination (*Q*_cπ_) to the adsorption of (**a**) Pb^2+^ and (**b**) Cd^2+^ onto BSL-BC. (**c**) Percentage release of elements (Mg^2+^, Na^+^, Ca^2+^, and K^+^) from BSL-BC into solution after adsorption of Pb^2+^ or Cd^2+^.

Abundant metal cations (e.g., K^+^, Na^+^, Ca^2+^, and Mg^2+^) typically retain on the biochar surface through electrostatic attraction and complexation with carboxyl and hydroxyl groups. These cations can exchange with Pb^2+^ and Cd^2+^ in solution and promote heavy metal adsorption^25^. As shown in Fig. 5c, significant amounts of K^+^ and Ca^2+^ were released from BSL-BC into solution after adsorption of Pb^2+^ and Cd^2+^. For the adsorption of Pb^2+^, 98% of K^+^ and Ca^2+^ were released into solution from BSL-BC, and 96% for adsorption of Cd^2+^; however, only a trace amount of Na^+^ was released and almost all Mg^2+^ was retained on BSL-BC. The release of Na^+^ and Mg^2+^ into solution accounted for 2% for adsorbed Pb^2+^ and 4% for adsorbed Cd^2+^ by BSL-BC.

#### Mineral precipitation

It was reported that anions (e.g., C_2_O_4_^2−^, CO_3_^2−^, PO_4_^3−^, and OH^−^) released from biochar may react with metal cations in solution to form mineral precipitates^20,51^. In this study, the XPS spectra identified the formation of Pb–O (Fig. 4g) and Cd–O (Fig. 4h) on BSL-BC surface after adsorption of Pb^2+^ and Cd^2+^, respectively. Consistently, the SEM–EDS analysis (Fig. S5) show scattered white granular crystals on the BSL-BC surface after adsorption process, with the spectra of elemental compositions confirming the presence of Pb^2+^ or Cd^2+^, P, C, and O on these crystals. These above characterization results indicate that precipitation of Pb(OH)_2_ and Cd(OH)_2_ may occur during adsorption.

According to the XRD analysis, CaCO_3_ and CaC_2_O_4_(H_2_O) crystals were present on the surface of BSL-BC before adsorption (Fig. S6a). After adsorption, PbC_2_O_4_ crystal was formed on the Pb-loaded BSL-BC (Fig. S6b), whereas CdCO_3_ and CdC_2_O_4_ crystals were identified on the Cd-loaded BSL-BC (Fig. S6c). These results are consistent with those aforementioned in analyzing the functional group complexation mechanisms. Although some previous studies^20,25,38^ reported the formation of Pb_3_(PO_4_)_2_ or Cd_3_(PO_4_)_2_ after biochar adsorption of Pb^2+^ or Cd^2+^, respectively, these precipitates were not present in the XRD pattern in this study, probably because they were below the detect limit of XRD.

#### Other potential mechanisms

Except for the above mechanisms, Pb^2+^/Cd^2+^-π coordination and electrostatic attraction also potentially contributed to the adsorption process. According to the FTIR analysis in Fig. S3, the peaks of –CH (700–900 cm^−1^), C=C (1318 cm^−1^), and C=O (1615 cm^−1^) on BSL-BC were reduced after adsorption of Pb^2+^ and Cd^2+^. These results indicate that Pb^2+^ and Cd^2+^ may interact with the π electrons during the adsorption process. Furthermore, determination of the PZC of BSL-BC suggested that its surface was neutral at pH 1.2 (Fig. S2). This indicates that at solution pH above 1.2, the surface of BSL-BC was negatively charged, which was favorable for adsorbing the Pb^2+^ and Cd^2+^ cations. Therefore, electrostatic attraction may occur that also contributed to the adsorption process.

#### Contribution of each adsorption mechanism

**Figure 6:**
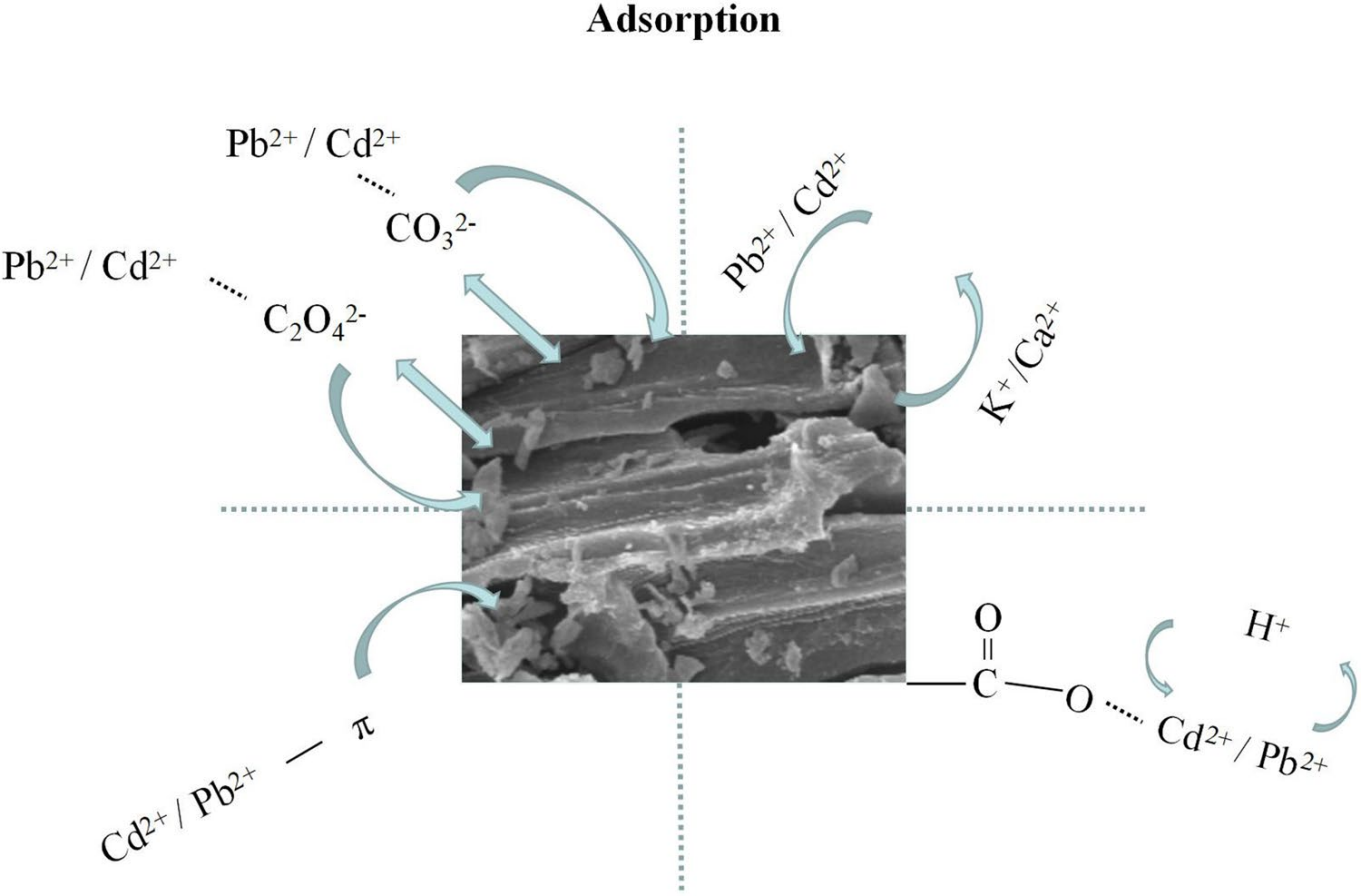
Mechanism of Pb^2+^ / Cd^2+^ adsorption onto BSL-BC.

The contribution of each adsorption mechanisms was calculated with Eqs. (12–16) and the results are presented in Fig. 5a,b. The schematic diagram of each adsorption mechanism is shown in Fig. 6. It is noted that electrostatic attraction was neglected during calculation of the mechanism contribution. The results show that mineral precipitation (*Q*_cmp_), metal ion exchange (*Q*_cme_), complexation with oxygen-containing functional group (*Q*_co_), and π-electron coordination (*Q*_cπ_) accounted for 88.8%, 8.4%, 0.1%, and 2.6%, respectively, for Pb^2+^ adsorption onto BSL-BC; and were 83.0%, 6.3%, 0.4%, and 10.4%, respectively, for Cd^2+^ adsorption. Therefore, mineral precipitation was the major adsorption mechanism that accounted for above 80% in both Pb^2+^ and Cd^2+^ adsorption.

The result is supported by the crystal formation of PbC_2_O_4_, CdCO_3_, and CdC_2_O_4_ on BSL-BC after adsorption as identified in the XRD spectra (Fig. S6). It is also consistent with literature studies. For instance, Wang et al.^20^ found that the dominant mechanism for Pb^2+^ adsorption by peanut shell biochar was mineral precipitation; similarly, Gao et al.^56^ also reported that the adsorption of Cd^2+^ by rice biochar was dominated by mineral precipitation, with relatively small contributions from complexation of Cd^2+^ with functional groups and coordination with π electrons on biochar.

## Conclusions

In this study, BSL-BC was successfully prepared from BSL as a recycling product from agricultural waste, with major physicochemical properties characterized. The synthesized BSL-BC exhibited strong adsorption for Pb^2+^ and Cd^2+^ in water. The adsorption rate data of Pb^2+^ and Cd^2+^ onto BSL-BC both followed the PSO kinetic model, indicating the process was chemisorption. The adsorption isotherm data of Pb^2+^ and Cd^2+^ could be well-described by the Langmuir and Freundlich models, respectively, whereas neither the Temkin or Dubinin–Radushkevich models provided satisfactory fitting results. The results indicate that monolayer and homogeneous adsorption occurred for Pb^2+^ onto BSL-BC, while the adsorption of BSL-BC for Cd^2+^ was multilayer and heterogeneous. The adsorption for Pb^2+^ and Cd^2+^ reached equilibrium after 8 h and 200 min, respectively, yielding maximum adsorption capacities of 302.2 and 32.03 mg/g. The optimum adsorption pH values were 5 and 8 for Pb^2+^ and Cd^2+^, respectively. Various characterization techniques show that the adsorption occurred via functional group complexation, cation exchange, mineral precipitation, π-electron coordination, and electrostatic attraction. Mineral precipitation was the major mechanism for the adsorption of Pb^2+^ and Cd^2+^ by BSL-BC, accounting for above 80% of mechanism contribution. This study provides a theoretical basis for recycling BSL waste to produce BSL-BC, which could be effectively applied in removal of Pb^2+^ and Cd^2+^ from contaminated water. Future study should examine the adsorption of BSL-BC for Pb^2+^ and Cd^2+^ under co-existing state or presented at lower metal concentrations in actual situation.

## Supplementary Information

Supplementary Information.

## Data Availability

The datasets generated during and/or analysed during the current study are available from the corresponding author on reasonable request.
