# Supplementary material for: Banana stem and leaf biochar as an effective adsorbent for cadmium and lead in aqueous solution
Source: Sci Rep. 2022 Jan 28;12:1584. doi: 10.1038/s41598-022-05652-7 (PMC8799728; doi:10.1038/s41598-022-05652-7)
Supplement: Supplementary file 1 — Supplementary Information. [file 41598_2022_5652_MOESM1_ESM.docx]

Supplementary Material for

**Banana stem and leaf biochar as an effective adsorbent for cadmium and lead in aqueous solution**

Xiyang Liu, Gaoxiang Li, Chengyu Chen*, Xiaorui Zhang Kuan Zhou, Xinxian Long*

*Guangdong Provincial Key Laboratory of Agricultural & Rural Pollution Abatement and Environmental Safety, College of Natural Resources and Environment, South China Agricultural University, 483 Wushan Road, Guangzhou**, Guangdong 510642, China*

*****Corresponding authors:

Xinxian Long, E-mail: [longxx@scau.edu.cn](mailto:longxx@scau.edu.cn)

Chengyu Chen, E-mail: [cychen@scau.edu.cn](mailto:cychen@scau.edu.cn)

**Contents:**

S1. Additional materials and methods (Pages S2-S3);

S2. Additional 4 tables (Pages S4-S7);

S3. Additional 6 figures (Pages S8-S13);

References (Page S14).

# S1. Additional materials and methods

## Optimum pyrolysis conditions for biochar

The pyrolysis conditions (temperature, residence time, and heating rate) played a key role in the physicochemical properties of biochar prepared from banana pseudo-stem and leaf (BSL-BC). Since these properties of BSL-BC are closely relate to its adsorption capacity for heavy metals, the optimum preparation conditions for BSL-BC were investigated in three sets of experiments as follows. (1) Temperature condition: The banana pseudo-stem and leave (BSL) biomass was treated at heating rates of 10 °C/min to 4 final temperatures (300, 400, 500, or 600 °C) and maintained for 2 h; (2) Residence time: The BSL biomass was heated to 400 °C at a rate of 5 °C/min and maintained for 4 residence times (1, 2, 3, or 4 h); (3) Heating rate: The BSL biomass was heated at 4 heating rates (5, 10, 15, or 15 °C/min) to the final temperature of 400 °C, which was maintained for 2 h.

The following Pb^2+^ adsorption experiments were conducted on the BSL-BC prepared at different pyrolysis conditions to investigate the optimum one. Firstly, 100 mg BSL-BC was added into 50 mL solution containing 200 mg/L of Pb^2+^ (pH = 5.0) in a 150 mL conical flask. It was shaken at 180 rpm and 25 °C for 8 h, after which the mixture was filtered with 0.30-0.50 μm Double Ring quantitative filter paper. The filtrate was measured for concentrations of Pb^2+^ and Cd ^2+^ by a flame atomic absorption spectrophotometer (AAS, Z-2300, Hitachi, Japan).

Table S1 shows that both the removal efficiency and adsorption amount (*Q*_e_) of Pb^2+^ by BSL-BC increased with higher pyrolysis temperature, which approached equilibrium at 400 °C, giving a Pb^2+^ removal efficiency of ~99%. Meanwhile, the biochar yield decreased from 55.14% to 31.42% as temperatures increased from 300 to 600 °C. Therefore, considering the cost and energy consumption, 400 °C was chosen as the optimum temperature for the production of BSL-BC based on the balance between biochar yield and adsorption performance.

For studying the effect of heating rate on biochar production, 5, 10, 15, and 20 °C/min were selected as experimental variables. Table S2 shows that the heating rate had insignificant influence on both removal efficiency and adsorption amount of Pb^2+^. The removal efficiency maintained at > 98% at heating rates of 10-20 °C/min, while the biochar yield achieved its maximum at 10 °C/min as 39.98%. Therefore, 10 °C/min was selected as the optimum heating rate for biochar production.

Table S3 shows that the removal efficiency of Pb^2+^ by BSL-BC slightly increased from 97.39% to 98.39% with increasing residence time from 1 to 4 h, while the biomass yields were 43.06%, 39.98%, 41.18%, and 37.86% at 1, 2, 3, and 4 h, respectively. Considering the energy consumption of practical application, the residence time of 3 h was chosen as for biochar production.

In summary, taking into considerations of both energy consumption and adsorption performance, the optimum pyrolysis condition for producing BSL-BC was chosen at a final temperature of 400 °C, heating rate of 10 °C/min, and residence time of 3 h.

# S2. Additional tables

**Table S1.** Effect of final pyrolysis temperature on BSL-BC yield and Pb^2+^ adsorption performance. The adsorption was conducted at initial Pb^2+^ concentration of 200 mg/L, adsorbent dosage of 2 g/L BSL-BC, pH 5.0, 25 ℃, and equilibrium time of 8 h.

| Temperature (℃) | Biochar yield (%) | Pb^2+^ removal efficiency (%) | *Q*_e_ (mg/g) ^a^ |
| --- | --- | --- | --- |
| 300 | 55.14 | 89.52 | 83.94 |
| 400 | 39.07 | 98.54 | 92.39 |
| 500 | 31.75 | 99.84 | 93.61 |
| 600 | 31.42 | 99.41 | 93.21 |

^a^ Adsorption amount of Pb^2+^ at equilibrium.

**Table S2.** Effect of heating rate on BSL-BC yield and Pb^2+^ adsorption performance. The adsorption was conducted at initial Pb^2+^ concentration of 200 mg/L, adsorbent dosage of 2 g/L BSL-BC, pH 5.0, 25 ℃, and equilibrium time of 8 h.

| Heating rate (℃/min) | Biochar yield (%) | Pb^2+^ removal efficiency (%) | *Q*_e_ (mg/g) ^a^ |
| --- | --- | --- | --- |
| 5 | 39.07 | 89.52 | 83.94 |
| 10 | 39.98 | 98.54 | 92.39 |
| 15 | 38.82 | 99.84 | 93.61 |
| 20 | 39.49 | 99.41 | 93.21 |

^a^ Adsorption amount of Pb^2+^ at equilibrium.

**Table S3.** Effect of residence time on BSL-BC yield and Pb^2+^ adsorption performance. The adsorption was conducted at initial Pb^2+^ concentration of 200 mg/L, adsorbent dosage of 2 g/L BSL-BC, pH 5.0, 25 ℃, and equilibrium time of 8 h.

| Residence time (h) | Biochar yield (%) | Pb^2+^ removal efficiency (%) | *Q*_e_ (mg/g) ^a^ |
| --- | --- | --- | --- |
| 1 | 43.06 | 97.39 | 96.81 |
| 2 | 39.98 | 98.28 | 97.69 |
| 3 | 41.18 | 98.88 | 98.29 |
| 4 | 37.86 | 98.39 | 97.81 |

^a^ Adsorption amount of Pb^2+^ at equilibrium.

**Table S4.** Comparison of maximum adsorption capacity (*Q*_m_) derived from the Langmuir isotherm model at room temperature for adsorption of Pb^2+^ and Cd^2+^ by BSC-BC with other biochar reported in the literature.

| Biochar | Raw material | Preparation temperature (℃) | pH | Dosage (g/L) | Time (min) | *Q*_m_ (mg/g) | | Reference |
| --- | --- | --- | --- | --- | --- | --- | --- | --- |
|  |  |  |  |  |  | Pb^2+^ | Cd^2+^ |  |
| BSL-BC | Banana stems and leaves | 400 | 5.00-5.50 | 1.0 | 480 | 302.2 | 32.03 | This study |
| B700 | Camellia seed husk | 700 | 5.00 | 1.0 | 1440 | 109.7 | 68.22 | Wu et al.^1^ |
| DM350 | Dairy manure | 350 | 5.18 | 5.0 | 600 |  | 51.40 | Xu et al.^2^  Wang et al.^3^ |
| PBC | Peanut shell | 350 | 5.00 | 4.0 | 2880 | 52.80 |  |  |
| MBC | Chinese medicine material residue | 400 | 5.00 | 4.0 | 2880 | 82.50 |  | Sun et al.^4^ |
| FBC | Rice husk | 600 | 5.00 | 2.5 | 1440 | 16.99 | 9.670 | Sun et al.^4^ |
| BC | Rape straw | 600 | 5.50 | 1.3 | 1440 |  | 32.74 | Li et al.^5^ |
| WSBC | Wheat straw | 600 | 5.00 | 5.0 | 60 | 100.00 | 19.72 | Trakal et al.^6^ |
| CSBC | Corn stalk | 300 | 6.00 | 5.0 | 360 | - | 33.94 | Ma et al.^7^ |
| BC | Banana peel waste | 600 | 5.50 | 1.0 | 30 | 263.0 | - | Amin et al.^8^ |

# S3. Additional figures

**
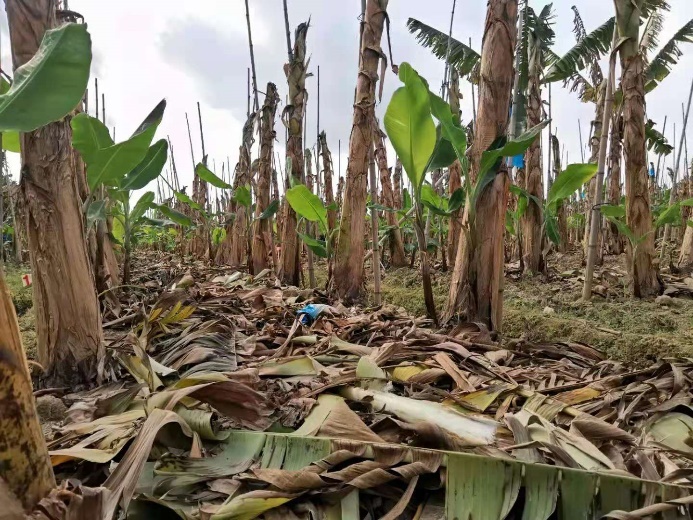
**
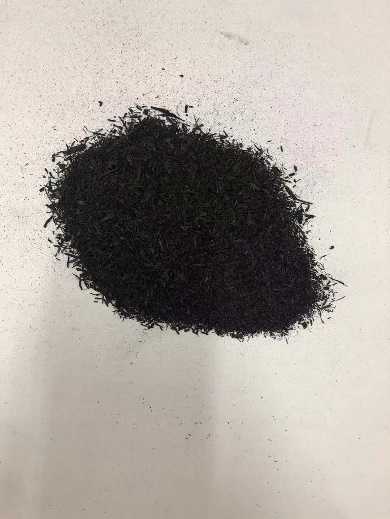


a

b

**Fig. S1.** Photos for (a) raw BSL and (b) BSL-BC synthesized under pyrolysis condition of 400 °C and 3 h.

**Fig. S2.** Zeta potential of BSL-BC as a function of in double-distilled water, yielding a point of zero charge (PZC) at pH 1.2. The experiments were conducted at a biochar to water ratio of 1:10000 (w:v) and at 25 °C.

**Fig. S3.** FTIR spectra of BSL-BC before (black) and after adsorption of Pb^2+^ (red) or Cd^2+^ (blue).







b

a

**Fig. S4.** SEM images for (a) BSL and (b) BSL-BC.





a


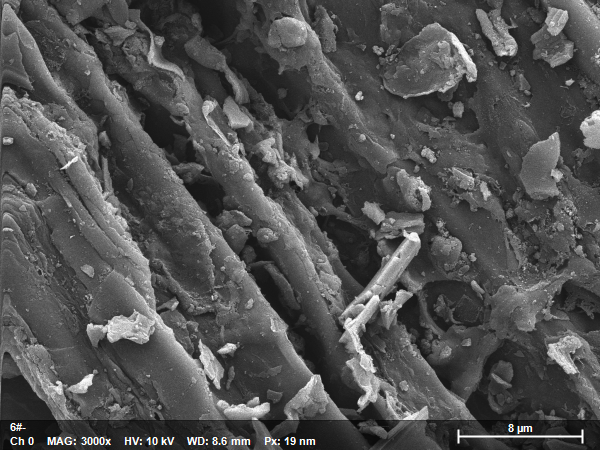


b


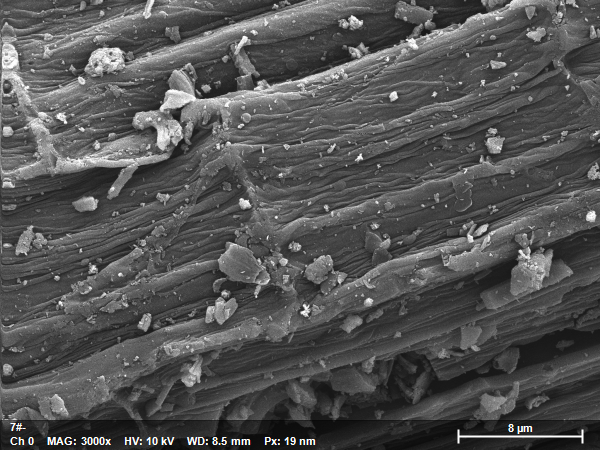


c

**Fig. S5.** SEM-EDS images of BSL-BC (a) before and after adsorption of (b) Pb^2+^ or (c) Cd^2+^.

a

b

c

**Fig. S6.** XRD images BSL-BC (a) before and after adsorption of (b) Pb^2+^ or (c) Cd^2+^.

**References**

1 Teng, D. et al. Efficient removal of Cd(II) from aqueous solution by pinecone biochar: Sorption performance and governing mechanisms. *Environ. Pollut.* 265, 115001, doi:10.1016/j.envpol.2020.115001 (2020).

2 Xu, X. et al. Removal of Cu, Zn, and Cd from aqueous solutions by the dairy manure-derived biochar. *Environ. Sci. Pollut. Res. Int.* 20, 358-368, doi:10.1007/s11356-012-0873-5 (2013).

3 Wang, Z. et al. Investigating the mechanisms of biochar's removal of lead from solution. *Bioresour. Technol.* 177, 308-317, doi:10.1016/j.biortech.2014.11.077 (2015).

4 Sun, C. et al. Enhanced adsorption for Pb(II) and Cd(II) of magnetic rice husk biochar by KMnO4 modification. *Environ. Sci. Pollut. Res. Int.* 26, 8902-8913, doi:10.1007/s11356-019-04321-z (2019).

5 Li, B. et al. Adsorption of Cd(II) from aqueous solutions by rape straw biochar derived from different modification processes. *Chemosphere* 175, 332-340, doi:10.1016/j.chemosphere.2017.02.061 (2017).

6 Trakal, L., Bingol, D., Pohorely, M., Hruska, M. & Komarek, M. Geochemical and spectroscopic investigations of Cd and Pb sorption mechanisms on contrasting biochars: engineering implications. *Bioresour. Technol.* 171, 442-451, doi:10.1016/j.biortech.2014.08.108 (2014).

7 Ma, F., Zhao, B. & Diao, J. Adsorption of cadmium by biochar produced from pyrolysis of corn stalk in aqueous solution. *Water Sci. Technol.* 74, 1335-1345, doi:10.2166/wst.2016.319 (2016).

8 Amin, M. T., Alazba, A. A. & Shafiq, M. Removal of Copper and Lead using Banana Biochar in Batch Adsorption Systems: Isotherms and Kinetic Studies. *Arab. J. Sci. Eng.* 43, 5711-5722, doi:10.1007/s13369-017-2934-z (2017).
